# From Lesion to Decision: AI for ARIA Detection and Predictive Imaging in Alzheimer’s Disease

**DOI:** 10.3390/biomedicines13112739

**Published:** 2025-11-10

**Authors:** Rafail C. Christodoulou, Platon S. Papageorgiou, Maria Daniela Sarquis, Ludwing Rivera, Celimar Morales Gonzalez, Daniel Eller, Gipsany Rivera, Vasileia Petrou, Georgios Vamvouras, Evros Vassiliou, Sokratis G. Papageorgiou, Michalis F. Georgiou

**Affiliations:** 1 Department of Radiology, Stanford University School of Medicine, Stanford, CA 94305, USA; deller@stanford.edu; 2 Department of Medicine, Medical School, National and Kapodistrian University of Athens, 11527 Athens, Greece; platopap@med.uoa.gr; 3 Department of Medicine, Universidad de Carabobo, Valencia 2001, Venezuela; mdsarquis58@gmail.com; 4 Department of Medicine, American University of Antigua College of Medicine, St. John’s 1451, Antigua and Barbuda; ludwingr@auamed.net (L.R.); celimarm@auamed.net (C.M.G.); gipsanyr@auamed.net (G.R.); 5 Department of Medicine, University of Ioannina, 45110 Ioannina, Greece; md07010@uoi.gr; 6 Department of Mechanical Engineering, National Technical University of Athens, 15772 Athens, Greece; gvamvouras@mail.ntua.gr; 7 Department of Biological Sciences, Kean University, Union, NJ 07083, USA; evassili@kean.edu; 8 1st Department of Neurology, Medical School, National and Kapodistrian University of Athens, Eginition Hospital, 15772 Athens, Greece; sokpapa@med.uoa.gr; 9 Department of Radiology, Division of Nuclear Medicine, University of Miami, Miami, FL 33136, USA

**Keywords:** Alzheimer’s disease, amyloid-related imaging abnormalities, ARIA-E, ARIA-H, artificial intelligence, MRI, anti-amyloid monoclonal antibodies, detection, prediction, imaging

## Abstract

**Background:** Alzheimer’s disease (AD) remains the leading cause of dementia worldwide, with anti-amyloid monoclonal antibodies (MABs) marking a significant advance as the first disease-modifying therapies. Their use, however, is limited by amyloid-related imaging abnormalities (ARIA), which appear as vasogenic edema or effusion (ARIA-E) and hemosiderin-related changes (ARIA-H) on MRI. Variability in imaging protocols, subtle early findings, and the lack of standardized risk models challenge detection and management. **Methods:** This narrative review summarizes current artificial intelligence (AI) applications for ARIA detection and risk prediction. A comprehensive literature search across PubMed, Embase, and Scopus identified studies focusing on MRI-based AI analysis, lesion quantification, and predictive modeling. **Results:** The evidence is organized into six thematic domains: ARIA definitions, imaging challenges, foundations of AI in neuroimaging, detection tools, predictive frameworks, and future perspectives. **Conclusions:** AI offers promising avenues to standardize ARIA evaluation, improve lesion quantification, and enable individualized risk prediction. Progress will depend on multicenter datasets, shared frameworks, and prospective validation. Ultimately, AI-driven neuroimaging may transform how treatment-related complications are monitored in the era of anti-amyloid therapy.

## 1. Introduction

Alzheimer’s disease (AD) is the most common cause of dementia worldwide, affecting over 55 million people, and represents a growing public health and socioeconomic challenge [1]. It is a progressive neurodegenerative disorder characterized by a gradual decline in memory, cognition, and functional abilities. The neuropathologic hallmarks of AD include extracellular amyloid β (Aβ) deposition forming senile plaques and intracellular neurofibrillary tangles composed of hyperphosphorylated tau protein [2]. The amyloid cascade hypothesis proposes that the pathological accumulation of Aβ peptides is the initiating event driving the disease process.

Specifically, Aβ42 is predominantly deposited in the brain parenchyma, whereas Aβ40 more frequently localizes within cerebral vessel walls, producing a synergistic effect on disease progression [3]. The aggregation of Aβ42 into soluble oligomers and insoluble fibrils disrupts synaptic signaling, alters calcium homeostasis, and induces oxidative stress, ultimately leading to neuronal dysfunction and death. In parallel, vascular deposition of Aβ40 contributes to cerebral amyloid angiopathy (CAA) by impairing vessel wall integrity and cerebral perfusion, further amplifying neurodegenerative damage [3]. The accumulation of Aβ triggers downstream pathological events, including tau hyperphosphorylation, microglial activation, and chronic neuroinflammation [4]. These processes promote synaptic loss, mitochondrial dysfunction, and progressive neuronal degeneration in hippocampal and cortical regions. Over time, the interplay between parenchymal and vascular amyloid burden accelerates both gray and white matter atrophy, underpinning the clinical syndrome of dementia observed in AD [5].

Neuroimaging is central in diagnosing dementia by identifying underlying pathologies and excluding reversible causes such as brain neoplasms [6]. Structural MRI provides critical insights into cortical and subcortical atrophy patterns that distinguish AD from other dementias, including frontotemporal dementia (FTD) and vascular dementia (VD) [7]. Functional modalities such as FDG-PET assess cerebral glucose metabolism and reveal hypometabolic signatures in the temporoparietal and posterior cingulate cortices, which are considered diagnostic hallmarks of AD. More recently, molecular imaging with amyloid PET and tau PET has enabled in vivo visualization of Aβ and tau deposits. This facilitates earlier and more accurate diagnosis, particularly atypical or prodromal presentations [8,9]. Beyond diagnosis, neuroimaging supports longitudinal disease staging and quantifies atrophy or hypometabolism to evaluate therapeutic responses in clinical trials for disease-modifying agents [7].

Despite these technological advances, diagnostic misclassification remains common in real-world clinical practice due to the biological heterogeneity of AD and variability in imaging interpretation [1]. As a result, translational research has increasingly focused on developing integrated diagnostic frameworks that combine neuroimaging with genetic, biomarker, and neuropsychological data to enhance individualized disease prediction and therapeutic decision-making. In parallel, neuromodulatory interventions such as non-invasive brain stimulation (NIBS) have shown promise in modulating cognition and mitigating neuropsychiatric symptoms associated with AD [10,11,12].

For decades, pharmacologic treatments for AD provided only symptomatic relief through cholinesterase inhibitors and NMDA receptor agonists, without altering disease trajectory. Recently, monoclonal antibodies (MABs) such as aducanumab, lecanemab, and donanemab have emerged as the first disease-modifying therapies targeting the amyloid cascade. These antibodies bind aggregated Aβ, facilitating its clearance via microglial activation and complement-mediated pathways. Clinical trials have demonstrated reduced amyloid plaque burden and modest slowing of cognitive decline in early-stage AD populations [13]. However, their therapeutic potential is constrained by a significant adverse effect known as amyloid-related imaging abnormalities (ARIA) [14]. ARIA represents a spectrum of treatment-emergent MRI findings that reflect increased vascular permeability following amyloid clearance. It is classified into vasogenic edema and/or effusion (ARIA-E) and hemosiderin-related abnormalities, including cerebral microhemorrhages and superficial siderosis (ARIA-H).

In Phase 3 trials, ARIA-E occurred in approximately 12.6% of lecanemab-treated participants, while ARIA-H was observed in about 16.0% [15]. In comparison, high-dose aducanumab treatment yielded ARIA-E rates approaching 35% and ARIA-H rates around 15–20% [16]. Although most cases are asymptomatic, some necessitate treatment interruption or discontinuation, and rare severe presentations may include confusion, encephalopathy, or seizures.

The diagnosis of ARIA relies primarily on MRI, with T2-FLAIR sequences detecting ARIA-E and susceptibility-weighted or T2*-GRE sequences identifying ARIA-H. Serial MRIs are recommended at baseline and during therapy to monitor safety [11]. Nevertheless, ARIA detection remains challenging even for experienced radiologists due to subtle imaging features, heterogeneity in MRI protocols, and inter-reader variability. Risk prediction is equally imprecise, as currently recognized factors such as APOE ε4 carriership, anticoagulant use, and a high baseline microbleed burden lack integration into standardized predictive models [17,18]. Consequently, there is a pressing need for automated, objective tools that can enhance diagnostic accuracy, support risk stratification, and facilitate consistent monitoring across centers.

Artificial intelligence (AI), encompassing deep learning and radiomic methodologies, offers a promising avenue to address these limitations [19]. Radiomics enables the extraction of high-dimensional quantitative features from MRI scans, capturing subtle textural and morphological characteristics invisible to human observers. When combined with genetic, clinical, or laboratory variables, these imaging-derived biomarkers can contribute to predictive models that personalize disease assessment and therapeutic safety [20].

Several studies have demonstrated the potential of AI in related neurological domains. Automated algorithms for detecting cerebral microbleeds on MRI have achieved pooled sensitivity, specificity, and accuracy of 91.6%, 93.9%, and 92.7%, respectively [21]. Similarly, radiomics-based approaches for edema prediction following stroke have achieved an area under the curve (AUC) of 0.94, with pooled sensitivity and specificity of 81.1% and 92.6%, respectively [22]. Convolutional neural network (CNN) models have identified microbleeds in traumatic brain injury with sensitivities up to 93% [23]. These findings highlight the strong performance and translational promise of AI-based detection systems for vascular and inflammatory imaging features.

Applying these approaches to ARIA monitoring may enhance early identification, quantification, and risk prediction. AI-based tools can reliably detect and characterize neuroimaging patterns such as edema and microbleeds, lesions central to ARIA surveillance. When integrated with patient-level data, including APOE genotype or baseline amyloid burden, AI models can generate individualized risk estimates, facilitating the safer administration of anti-amyloid therapies [24].

In this narrative review, we synthesize current knowledge on ARIA and emphasize the emerging role of AI in its detection and risk prediction. To our knowledge, based on a comprehensive literature search conducted on 13 August 2025, this is the first review dedicated to AI applications for ARIA. By integrating evidence from neuroimaging, artificial intelligence, and clinical trial data, we aim to: (i) provide a comprehensive overview of the field; (ii) highlight methodological advances and ongoing challenges in AI-based ARIA assessment; and (iii) discuss translational implications for improving patient selection, treatment monitoring, and safety. This work bridges clinical, imaging, and computational perspectives, underscoring the potential of AI-driven neuroimaging to advance precision medicine in AD.

## 2. Discussion

Amyloid-related imaging abnormalities (ARIA) represent the principal radiological safety concern associated with anti-amyloid monoclonal antibodies. Understanding their imaging characteristics, underlying mechanisms, and emerging detection strategies is essential for optimizing therapeutic safety. This study integrates radiological, clinical, and AI perspectives to contextualize ARIA in current AD research, examine recent methodological advances, and identify key translational challenges.

**Figure 1:**
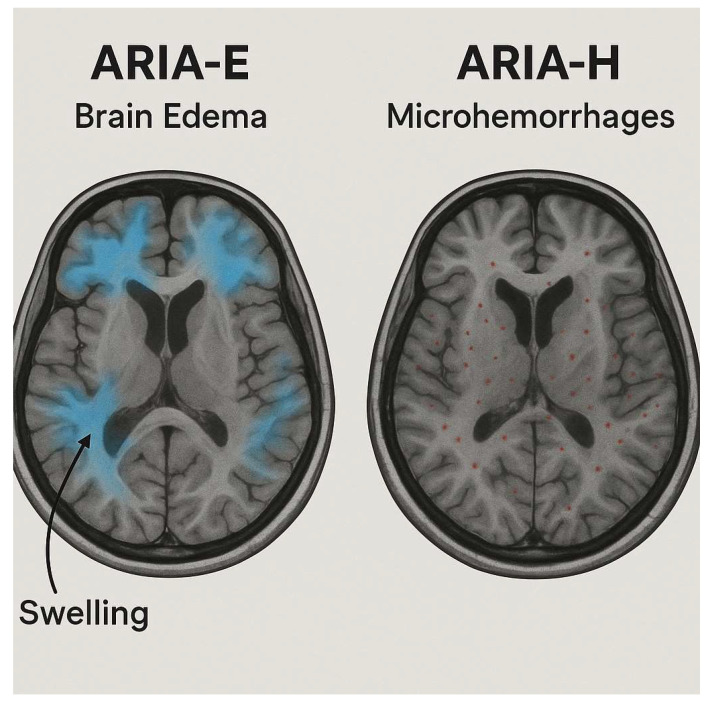
Representative MRI scans showing ARIA. On the left, ARIA-E indicates brain edema characterized by areas of swelling (highlighted in blue). On the right, ARIA-H displays microhemorrhages marked by small red dots throughout the brain. Created in BioRender. Christodoulou, R. (2025) https://BioRender.com/801nbt2 (accessed on 2 September 2025).

ARIA is predominantly an imaging-based phenomenon, and the precise pathophysiological mechanism is underexplored. The hypothesis suggests that the binding of MABs in the parenchyma and vascular amyloid plaques results in loss of the vessel wall integrity and, therefore, leaks of proteinaceous fluid (ARIA-E) and hemeproducts (ARIAA-H) [Figure 1] [25]. ARIA-E involves vasogenic edema or sulcal effusion, seen as hyperintense on T2 FLAIR. Increased endothelial permeability to serum proteins mainly affects the occipital and parietal lobes. The Alzheimer’s Association Work Group established this terminology, which has since been adopted in major anti-amyloid trials [11]. From an imaging perspective, ARIA-E often presents as asymmetric cortical-subcortical hyperintensity with or without mass effect [17]. At the same time, ARIA-H includes hemosiderin changes, cerebral microhemorrhages (CMBs), and superficial siderosis seen on SWI or GRE sequences. Microbleeds cause hypointensities, while superficial siderosis is linear cortical signal loss [17,26]. These findings form the basis for ARIA-E and ARIA-H diagnosis, but standardized definitions were needed for consistency in trials [Table 1].

Imaging criteria for ARIA are delineated in pivotal trials such as CLARITY-AD (lecanemab), where ARIA-E was defined as new or increased FLAIR hyperintensity consistent with vasogenic edema or sulcal effusion. ARIA-H was described as new microhemorrhages, macrohemorrhages, or superficial siderosis seen on SWI/GRE [28]. In TRAILBLAZER-ALZ2 (donanemab), the same lesion definitions were employed with ARIA adjudicated by neuroradiologists as part of the trial’s safety review [29]. These trial-based criteria guided safety monitoring on drug development and informed the post-approval clinical management strategies outlined in Appropriate Use Recommendations.

AURs for lecanemab and donanemab provide clinical management guidelines. The lecanemab baseline MRI should be performed within 12 months, with repeated scans before infusions at 5, 7, and 14 weeks and at week 52 in high-risk patients [30]. Donanemab guidance recommends a baseline MRI within 12 months, excluding patients with >4 microbleeds or any superficial siderosis and MRIs before the 2nd, 3rd, 4th, and 7th doses, plus a 12th dose scan in high-risk individuals [31]. Both sets of AURs advise dose interruption or discontinuation for symptomatic ARIA or severe asymptomatic cases [32]. Applying these definitions and management protocols in large phase 3 trials has provided valuable data on ARIA’s frequency, timing, and resolution patterns in AD patients.

ARIA’s incidence and natural history vary by drug, dose, and patient genotype. In the CLARITY-AD clinical trial, ARIA-E occurred in 12.6% of lecanemab-treated patients in the core study, while ARIA-H occurred in 16.0% [28,33]. TRAILBLAZER-ALZ 2 trial reported ARIA-E in 24% and ARIA-H in 31% of donanemab-treated participants [29]. Events often occurred in the first 3–6 months, typically resolved with conservative management, while recurrence was uncommon [33]. Although these findings have refined the epidemiological understanding of ARIA, their detection and monitoring still rely heavily on MRI, where technical variability continues to limit reproducibility across studies.

ARIA detection hinges on a routine MRI tailored to the lesion type: FLAIR for edema/effusion (ARIA-E) and SWI/GRE for hemosiderin signs (ARIA-H) [34]. SWI and Quantitative Susceptibility Mapping (QSM) are critical for the detection, but MRI acquisition parameters also directly influence lesion visibility in the final assessment [35]. However, the reliability of these methods is highly influenced by acquisition parameters, such as echo time, slice thickness, and magnetic-field strength, which vary widely among scanners and institutions. For example, a center using shorter TE, thicker slices, and low resolution may underreport ARIA-H, while others using long TE, high resolution, and 3T or even 7T MRI scanners may detect more lesions in the same patient population [26]. Deep SWI, a recent AI-enhanced susceptibility approach, claims to improve image quality without extra time and support more reliable microbleed visibility [36]. Classical and deep learning microbleed detectors built specifically on SWI show strong feasibility in adaptation to radiological workflows. While the first is time-efficient due to optimization, it has the trade-off of false positives [37]. Deep learning models can minimize the false positive amount by applying high-end filters that enable the differentiation of diamagnetic products (Ca^2+^) from paramagnetic microbleeds. Notably, Liu et al. (2019) developed a 3D SWI deep learning model that achieves a sensitivity of 95.8% and minimizes false positive microbleeds to 1.5 per case [37]. Complementary methods QSMnet expand the susceptibility toolbox relevant to ARIA-H by providing high-quality QSM maps from conventional MRIs, which can be helpful in the quantification of ARIA-H lesions [38]. These advances illustrate how AI can enhance lesion visibility and harmonize assessments in multicenter anti-amyloid therapy trials.

Despite these improvements, accurate diagnosis still requires integration of multiple MRI sequences. DWI(Diffusion Weighted Imaging) serves as an essential adjunct to exclude an acute infarct when new FLAIR changes are encountered in AD patients with ARIA-E [27]. This step is crucial for ARIA-E, where vasogenic edema from anti-amyloid treatments can mimic or be mimicked by ischemic changes. On DWI, ARIA-E lesions lack diffusion restriction, helping distinguish them from acute infarction, which shows a high DWI signal and low apparent diffusion coefficient (ADC) values. Therefore, DWI and ADC increase diagnostic confidence and support timely, appropriate management decisions without unnecessary treatment interruption. However, the reliability of such assessments depends heavily on the quality and consistency of MRI acquisition across institutions. Variability in sequence availability, parameter settings, and image interpretation can still compromise diagnostic accuracy and reproducibility in real-world practice.

Differences in acquisition protocols, slice thickness, and b values can influence lesion conspicuity and lead to misclassification, either overcalling ARIA-E as a stroke or missing subtle vasogenic edema [39]. General variability in field strength, echo times, voxel size, and SWI implementations results in site-to-site inconsistencies and complicates longitudinal reads [35]. Even senior radiologists show inter-observer differences for subtle microbleeds and borderline edema [40,41]. Underreporting is common in busy radiology workflows, especially for tiny cortical lesions on low-signal-to-noise-ratio images. Augmentation studies indirectly have demonstrated that this underreporting is partly rooted in data scarcity and imaging artifacts [42]. Most medical centers’ databases lack subtle or artifact-rich examples due to the post-processing techniques employed. Therefore, radiologists have fewer opportunities to distinguish them. Motion and susceptibility artifacts can mimic microbleeds, leading to false positives, while noise can mask true lesions, causing false negatives [43]. The current gap highlights the need for standardization and robust imaging pipelines to harmonize site detection. Addressing these challenges requires methods that can harmonize imaging data and reduce inter-observer variability.

AI-assisted standardized detection is a promising development, driven by advances in machine learning and deep learning. For instance, two-stage pipelines can first localize candidate lesions and filter out false positives; end-to-end SWI-based networks can be optimized to recognize ARIA-H patterns across protocols consistently; and susceptibility-contrast enhancement tools can normalize input data before analysis [36,44,45]. Such methods combine coordinated data collection with automated analysis, enabling reproducible ARIA detection across multiple centers. AI in neuroimaging depends on several well-established methods.

CNNs are tools for image classification and segmentation. U-Net and its self-configuring version, nnU-Net, are standard frameworks for voxel-wise lesion mapping in MRI tasks [46,47]. Borrowed from language modeling, transformers have demonstrated potential for incorporating long-range spatial context, while radiomics pipelines extract hundreds of quantitative features from routine MRI for traditional machine-learning models [48]. These architectures have been refined and validated across various neuroimaging datasets, providing robust building blocks for new applications such as ARIA detection. Their proven success in analogous MRI tasks, where lesion morphology and imaging characteristics overlap with those of ARIA, offers a clear opportunity for transfer learning.

**Figure 2:**
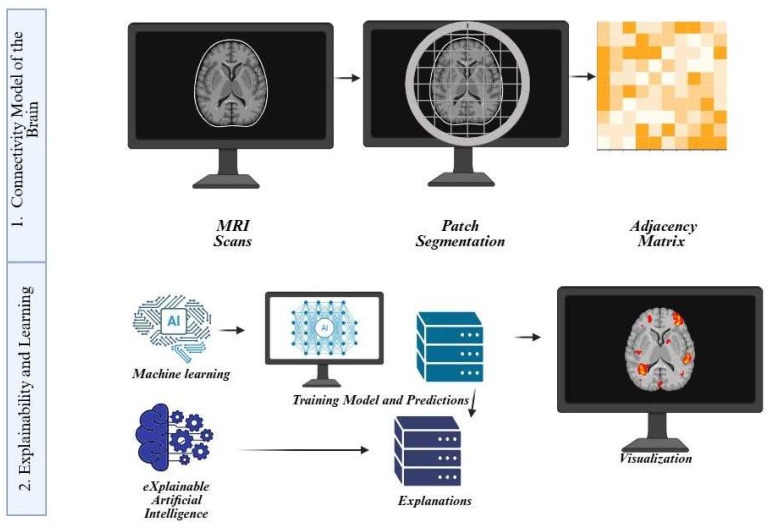
General workflow of AI in neuroimaging. This schematic illustrates the key stages of applying AI to brain MRI analysis. In panel (**1**), structural MRI scans are divided into image patches, which are then converted into adjacency matrices to model brain connectivity. In panel (**2**), machine learning and explainable AI (XAI) approaches are shown, where predictive models are trained on imaging data to generate outcomes. At the same time, explainability methods highlight the most informative brain regions and provide interpretable visualizations. Created in BioRender. Christodoulou, R. (2025) https://BioRender.com/6ibbhx3 (accessed on 2 September 2025).

In acute stroke, CNN and Unet-based networks achieve rapid and accurate segmentation of vasogenic edema and infract core on FLAIR/DWI [41,49]. Similarly, in brain tumors, nnU-Net pipelines consistently delineate peritumoral edema on MRI in the BraTS challenge [50], a task morphologically similar to ARIA-E on FLAIR. Furthermore, CAA applications of high-sensitivity deep learning models on SWI/GRE range from candidate generation (for recommendation systems) to the suppression of false positives in microbleed detection [41,45,51,52]. These applications overlap with ARIA types, as ARIA-E indicates vasogenic edema, and ARIA-H microbleeds resemble CAA. Studies show transfer learning enables CNN models to adapt to new brain-MRI tasks with limited data. These findings support using pre-trained, general architectures as effective starting points for accelerating ARIA tool development by leveraging pre-trained weights and augmentation strategies to adapt to amyloid-treated populations [53,54]. The general workflow of AI in neuroimaging, including connectivity modeling, predictive pipelines, and explainable AI visualization, is illustrated in [Figure 2].

Building upon these general AI frameworks, recent efforts have produced dedicated platforms designed explicitly for ARIA detection and quantification. The advanced ARIA-specific platform, icobrain aria (Icometrix), automates quantification of ARIA-E and ARIA-H on paired baseline follow-up MRI. It performs registration, CNN segmentation, and quantification, reviewing lesion counts, volumes, and overlays [24,55,56]. In a study with 16 neuroradiologists interpreting 199 MRI pairs from anti-amyloid trials, AI assistance improved performance and inter-reader agreement. Each MRI pair, comprising baseline and follow-up scans, helps detect new or worsening lesions, excluding stable findings. This approach mirrors trial ARIA definitions, improving specificity by removing pre-existing abnormalities and sensitivity for subtle lesions via subtraction and change-mapping. Assisted AUCs range from 0.82 to 0.87 for ARIA-E and 0.78 to 0.83 for ARIA-H, with sensitivity from 71% to 87% and 69% to 79%, maintaining ≥80% specificity [24] [Table 2].

**Figure 3:**
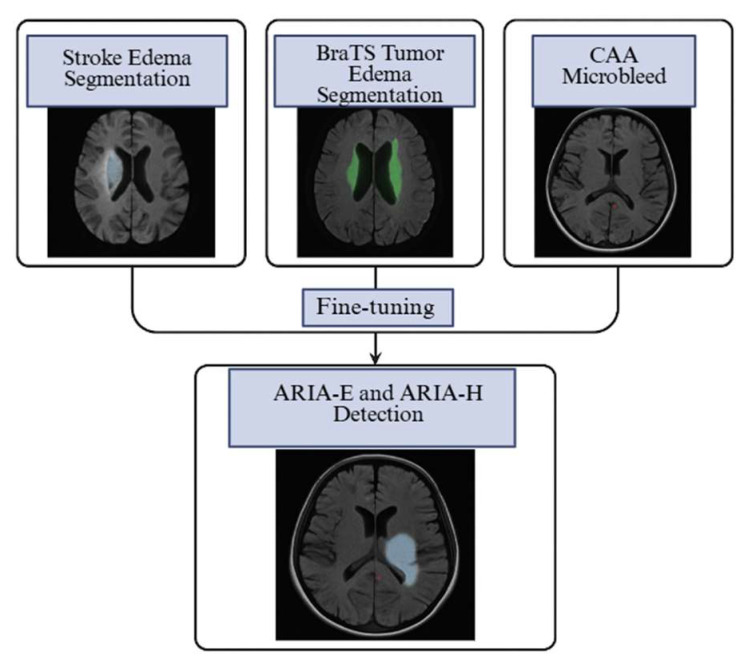
Transfer Learning in ARIA Detection. Created in BioRender. Christodoulou, R. (2025) https://BioRender.com/guhh2ap (accessed on 2 September 2025).

Following these promising validation results, subsequent studies have focused on clinical translation and regulatory approval to enable broader deployment of such tools in practice. Additional reports describe its clinical validation on proprietary trial datasets (e.g., EMERGE, ENGAGE, PRIME) and its FDA 510(k) clearance in 2024 for ARIA detection and quantification [57,58]. Similar software by Cortechs.ai https://www.cortechs.ai/ (accessed on 2 September 2025), NeuroQuant ARIA 5.0, received similar clearance in late 2024, offering a second commercial option that quantifies ARIA-E on FLAIR and ARIA-H on GRE/SWI [59]. These regulatory milestones underscore the growing clinical maturity of AI-based ARIA assessment, shifting the discussion from technical feasibility to real-world integration. Neuroradiologists’ perspectives emphasize that AI assistance increases sensitivity, particularly for subtle ARIA. Still, they may slightly reduce specificity, supporting a busy workflow in which AI overlays complement standardized acquisition and radiologist review of AI output to enhance safety monitoring in patients receiving anti-amyloid therapies [60] [Figure 3]. These AI-assisted workflows extend beyond diagnostic support to inform clinical decision-making and risk-stratification strategies by enhancing lesion detection and standardizing image interpretation.

**Figure 4:**
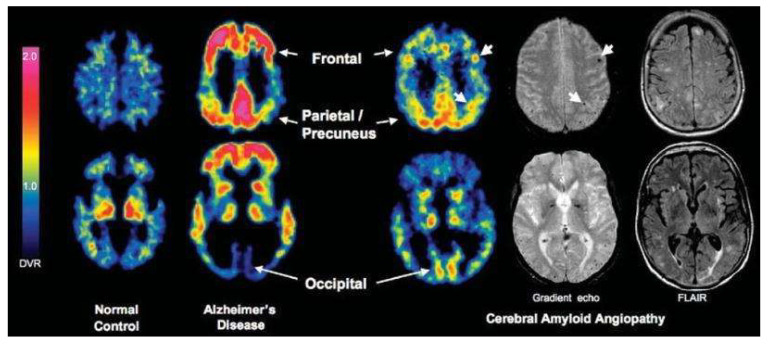
Multimodal imaging biomarkers relevant to ARIA risk prediction. PET demonstrates amyloid burden in Alzheimer’s disease, while GRE and FLAIR MRI show microhemorrhages and white matter hyperintensities. When combined with APOE ε4 status and treatment factors, these imaging markers contribute to ARIA risk stratification. Created in BioRender. Christodoulou, R. (2025) https://BioRender.com/xcro709 (accessed on 2 September 2025).

ARIA remains the primary safety concern for anti-amyloid monoclonal antibodies, affecting eligibility, dose adjustments, and MRI monitoring. Accurate risk prediction enables clinicians to identify high-risk patients before treatment, customize dosing regimens, and adjust follow-up imaging intervals [11,61]. Evidence from post hoc analyses of key trials and expert guidelines highlights clinical, genetic, and imaging markers as predictors [18,34]. The most robust predictor is the APOE ε4 genotype, with ε4/ε4 homozygotes showing the highest ARIA incidence across trials [18,30,34,62]. Baseline MRI findings, especially microhemorrhages and white matter hyperintensities, are also significant; in the CLARITY-AD trial, these features and APOE ε4 status independently predicted ARIA-E [15]. Anticoagulant use increases symptomatic hemorrhage risk, prompting cautions [16]. Higher baseline amyloid plaque burden on PET also correlates with ARIA risk, as shown in donanemab analyses [29]. Dose, APOE ε4, amyloid burden, microhemorrhages, white matter abnormalities, and anticoagulant use form the key risk profile [25] [Table 3, Figure 4].

Unfortunately, ARIA prediction models are rare, mostly regression-based, with the CLARITY-AD model providing interpretable risk scores for APOE ε4, microhemorrhages, and white matter hyperintensities [15]. Machine learning can personalize estimates by integrating data like genetics, MRI, PET, and treatment, enabling real-time risk updates via AI detection of lesions, volumetrics, and regional data. However, translating these conceptual advances into robust predictive models remains challenging. ARIA events, while clinically significant, are relatively uncommon for some agents and regimens, with ARIA-E rates ranging from <10% in particular phase 2 lecanemab cohorts to ~20–24% in phase 3 trials, and clustering early during titration [15,63,64]. This rarity creates data scarcity and class imbalance, which are recognized barriers to robust model training and generalization in medical imaging AI [65].

Furthermore, domain shift caused by variations in MRI acquisition parameters, field strength, and susceptibility imaging protocols between trials and real-world settings can limit the portability of models trained on harmonized datasets [66]. Tackling these issues will involve developing multicenter, harmonized ARIA datasets and potentially using transfer learning techniques to adapt models to different imaging environments. In parallel with these technical efforts, a complementary line of research focuses on leveraging AI’s multimodal capabilities to integrate imaging, biomarkers, and clinical variables. AI can improve diagnostic accuracy, enable earlier detection, and provide consistent evaluations across centers in real-world clinical practice [67]. Emerging biomarker research, including studies connecting cerebrospinal erythrocyte load with hippocampal atrophy [68] and showing its interaction with phosphorylated tau in speeding up entorhinal degeneration [69], highlights the kinds of biomarkers that could be integrated into future AI-based models. Recent studies underscore AI’s growing role in diagnosing and managing AD. Diagnostic frameworks confirm that AI tools can support early and accurate AD detection in clinical settings [70]. A biomarker-based AI model demonstrated high reliability in differentiating Mild Cognitive Impairment (MCI) from normal cognition, with the advantage of abstaining in borderline cases to minimize misclassification [71]. AI applications in PET and MRI imaging highlight the transformative impact of multimodal pipelines on diagnosis, prognosis, and staging, allowing a more personalized approach [67]. These findings underscore AI’s capacity to unify diverse biomarkers within a single analytical framework. Consequently, AI is also expanding to monitor treatment responses and improve the prediction of ARIA risk. However, results reveal potential and current limitations in deploying AI-assisted ARIA detection and risk assessment in everyday practice. Enhancing sensitivity to detect subtle lesions and incorporating multiple risk factors could significantly improve patient selection, dosing, and safety oversight, especially with anti-amyloid treatments [56,57]. However, most existing models depend heavily on single datasets, often from tightly controlled clinical trials, which restricts their applicability in varied real-world settings due to generalizability concerns. Addressing this gap explicitly is crucial, as meaningful clinical impact will require validation across multiple centers, standardized imaging protocols, and access to representative real-world data. Although the potential of AI is evident, significant practical barriers persist. Despite the advances in AI-assisted ARIA detection, progress is restricted by the absence of large, openly accessible, and richly annotated datasets. Most imaging data are from tightly controlled phase 2 and 3 anti-amyloid antibody trials [11,16,63].

Real-world datasets are usually small, inconsistently labeled, and often lack paired baseline–follow-up MRI sequences needed to track lesions over time. Furthermore, subtype representation has a marked imbalance, with mild ARIA-E dominating. At the same time, ARIA-H and severe ARIA-E cases are underrepresented, limiting model sensitivity for rare but clinically meaningful phenotypes. Significant domain shift results from variability in MRI field strength, coil type, SWI protocols, and post-processing, affecting lesion visibility, especially for subtle microbleeds and edema [33,72,73]. Also, differences in echo time, voxel resolution, and GRE implementation can alter detection accuracy, especially for subtle microbleeds [41,42]. A common problem in ML datasets is class imbalance, which biases models toward negative or mild findings, reducing sensitivity for rare events. Solutions include protocol harmonization, robust pre-processing, and transfer learning or domain adaptation [51,74]. Beyond these technical and data considerations, the path toward clinical deployment introduces additional regulatory and ethical dimensions. Before clinical use, AI systems need multicenter validation and regulatory approval. CE Mark clearances for ARIA detection show feasibility but highlight the extensive verification process. Agencies emphasize explainability, like lesion overlays and summaries, to keep clinicians’ trust. At the same time, data laws like HIPAA and GDPR limit centralized data sharing, boosting interest in federated AI training. Medico-legal accountability for AI ARIA reports remains uncertain, underscoring the need for human oversight and clear responsibility frameworks. To address these challenges, collaborative initiatives will be essential.

The field should aim to develop multicenter ARIA research collaborations to create standardized datasets for large-scale training and validation. Utilizing public datasets and challenges could increase transparency and foster innovation. Adding AI lesion maps, volumetric data, and change metrics into reports may improve decision-making and MRI. Prospective trials testing AI-assisted monitoring, including diagnostic accuracy, safety, and workflow assessments, are essential for clinical validation.

As expected, this study has some limitations. First, much of the current evidence on AI for ARIA detection and risk prediction comes from proprietary clinical trial datasets or small, single-center studies. This reliance limits how well the findings apply to real-world clinical settings, where MRI protocols, patient demographics, and lesion prevalence vary significantly. Second, the lack of large, publicly available, fully annotated ARIA datasets has prevented formal meta-analysis. As a result, this review is based on qualitative synthesis, which might not entirely reflect direct performance comparisons between AI tools or imaging protocols.

## 3. Conclusions

Anti-amyloid MABs represent a significant therapeutic milestone in AD, offering the first disease-modifying effect through amyloid clearance. However, their clinical implementation remains constrained by the risk of amyloid-related imaging abnormalities (ARIA), whose subtle MRI manifestations and variable acquisition parameters make reliable detection and risk prediction difficult. AI, through radiomics, DL, and techniques such as multimodal data fusion, provides a pathway to overcome these obstacles by standardizing ARIA assessment, quantifying lesion burden, and integrating genetic, imaging, and clinical data for individualized safety profiling.

Recent FDA-cleared AI platforms demonstrate the feasibility of automated ARIA quantification and improved reader performance, underscoring the field’s growing translational maturity. However, validation remains restricted to proprietary datasets, limiting reproducibility and generalizability to external populations. Future work should focus on multicenter collaborations to develop harmonized, open-source ARIA repositories and on prospective trials evaluating the real-world clinical impact of AI-assisted monitoring on treatment safety, dosing, and outcomes. Advancing these efforts will solidify AI’s role as a critical enabler of precision medicine in AD therapy, ensuring safer and more personalized delivery of disease-modifying treatments.

## Figures and Tables

**Table 1:** ARIA types, imaging features, and detection protocols.

Study	ARIA Type	Pathophysiology	Preferred MRI Sequence(s)	Typical Locations	Radiologic Signs	Trial-Based Definition (Examples)	Key Risk Factors	Key Clinical Impact	Reference
Roytman et al., 2023	**ARIA-E** (edema/effusion)	Vasogenic edema or sulcal effusion from increased vascular permeability after amyloid clearance	T2-FLAIR ± DWI (to exclude infarct)	Occipital and parietal lobes	Hyperintense cortical-subcortical signal, often asymmetric; may have mild mass effect	CLARITY-AD (2022): New or increased FLAIR hyperintensity consistent with vasogenic edema/sulcal effusion; TRAILBLAZER-ALZ2 (2023): Similar definitions, neuroradiologist adjudicated	APOE ε4 homozygosity, higher amyloid burden, high-dose regimens	May require temporary dose interruption or modification; linked to symptomatic confusion or headache in rare cases	[25]
Jeong et al., 2023	**ARIA-H** (hemosiderin-related)	Microhemorrhages or superficial siderosis from vessel wall fragility after plaque clearance	SWI or GRE ± QSM (for quantification)	Cortical/subcortical regions, superficial sulci	Punctate hypointensities (microbleeds), linear cortical hypointensity (siderosis)	CLARITY-AD (2022): New micro/macrohemorrhages or superficial siderosis on SWI/GRE; TRAILBLAZER-ALZ2 (2023): Same lesion criteria, neuroradiologist adjudicated	APOE ε4, baseline microbleeds, anticoagulant use	Increases risk of symptomatic hemorrhage; may influence eligibility and anticoagulation management	[27]

**Table 2:** The Role of Transfer Learning in AI Models for ARIA Detection.

Study	Source Domain/Pretrained Model	Target Task (ARIA-Related)	Imaging Modality	Transfer Learning Strategy	Reported Performance in Analogous Task	Potential Benefits for ARIA Detection	Limitations/Considerations	Reference
Isensee et al., 2021	CNN models for acute stroke lesion segmentation (FLAIR/DWI)	ARIA-E segmentation	T2-FLAIR	Fine-tuning encoder–decoder weights on ARIA-E FLAIR data	Dice ≥ 0.85 in stroke edema segmentation	Captures vasogenic edema morphology; reduces need for large ARIA-E datasets	Edema appearance may differ in location and intensity between stroke and ARIA	[47]
Beheshti et al., 2025	nnU-Net trained for peritumoral edema in brain tumors (BraTS challenge)	ARIA-E detection	T2-FLAIR	Full-network retraining with ARIA cases + augmentation	Top BraTS scores for peritumoral edema	Handles diffuse cortical-subcortical hyperintensities; strong generalization	Tumor edema more heterogeneous than ARIA-E; requires domain adaptation	[48]
Hsu et al., 2023	SWI-based deep learning models for cerebral microbleed detection in CAA	ARIA-H microbleed detection	SWI/GRE	Transfer last layers; augment with ARIA-H SWI	Sensitivity 93–96%, FP ~1.5/case	Similar lesion morphology;robust small-lesion detection	Must adjust for distribution differences (location, number) between CAA and ARIA-H	[35]
Shafieioun et al., 2025	Radiomics models predicting cerebral edema post-stroke	ARIA-E risk prediction	T2-FLAIR ± DWI	Feature selection + retraining classifier	AUC 0.94 for edema prediction	Integrates imaging and clinical features; interpretable	Requires harmonized imaging features; small ARIA datasets may limit stability	[22]
Yoon et al. (2018)	MRI-QSM enhancement models (QSMnet) trained on susceptibility mapping	ARIA-H quantification	SWI/QSM	Use as preprocessing stage for susceptibility normalization	Improved microbleed conspicuity in QSM	Reduces domain shift in susceptibility protocols	Needs QSM acquisition or synthetic generation for deployment	[38]

**Table 3:** Incidence and timing of ARIA in major anti-amyloid trials.

Drug	Trial	Dose Regimen	*N* (Treatment Arm)	ARIA-E Incidence	ARIA-H Incidence	Median Onset	Resolution Rate	Recurrence	Notes
**Lecanemab**	CLARITY-AD (2022)	10 mg/kg biweekly	898	12.6%	17.3%	3–6 months	Most resolved with monitoring	Rare	Higher rates in APOE ε4 carriers; MRI at 5, 7, 14 weeks recommended for high-risk patients
**Donanemab**	TRAILBLAZER-ALZ2 (2023)	Titration to high dose	860	24%	31%	3–6 months	Most resolved	Rare	Modified titration in TRAILBLAZER-ALZ6 reduced ARIA-E while maintaining efficacy
**Aducanumab**	EMERGE/ENGAGE (2020)	10 mg/kg high dose	1105 combined High dose treatment group	Up to 35%	15–20%	Mostly in first 8 doses	Most resolved	Uncommon	Discontinuation recommended for severe or symptomatic cases; careful monitoring in APOE ε4 carriers

## Data Availability

Not applicable.

## Abbreviations

The following abbreviations are used in this manuscript:

AD	Alzheimer’s Disease
AI	Artificial Intelligence
APOE	Apolipoprotein E
Aβ	Amyloid-beta
ARIA	Amyloid-Related Imaging Abnormalities
ARIA-E	Amyloid-Related Imaging Abnormalities—Edema/Effusion
ARIA-H	Amyloid-Related Imaging Abnormalities—Hemosiderin-related
AUC	Area Under the Curve
BraTS	Brain Tumor Segmentation challenge
CAA	Cerebral Amyloid Angiopathy
CC BY	Creative Commons Attribution
CNN	Convolutional Neural Network
DWI	Diffusion-Weighted Imaging
FLAIR	Fluid-Attenuated Inversion Recovery
FP	False Positive
GRE	Gradient-Recalled Echo (MRI sequence)
IoMT	Internet of Medical Things
MAB(s)	Monoclonal Antibody(/ies)
MRI	Magnetic Resonance Imaging
NMDA	N-methyl-D-aspartate
QSM	Quantitative Susceptibility Mapping
SWI	Susceptibility Weighted Imaging
QSMnet	Quantitative Susceptibility Mapping neural network

## References

[1] Scheltens P., De Strooper B., Kivipelto M., Holstege H., Chételat G., Teunissen C.E., Cummings J., van der Flier W.M. (2021). Alzheimer’s disease. Lancet.

[2] McDade E.M. (2022). Alzheimer Disease. Continuum.

[3] Ma C., Hong F., Yang S. (2022). Amyloidosis in Alzheimer’s Disease: Pathogeny, Etiology, and Related Therapeutic Directions. Molecules.

[4] Lacosta A.-M., Insua D., Badi H., Pesini P., Sarasa M. (2017). Neurofibrillary Tangles of Aβx-40 in Alzheimer’s Disease Brains. J. Alzheimer’s Dis..

[5] Bondi M.W., Edmonds E.C., Salmon D.P. (2017). Alzheimer’s Disease: Past, Present, and Future. J. Int. Neuropsychol. Soc..

[6] Raji C.A., Benzinger T.L.S. (2022). The Value of Neuroimaging in Dementia Diagnosis. Continuum.

[7] Masdeu J.C. (2020). Neuroimaging of Diseases Causing Dementia. Neurol. Clin..

[8] Del Sole A., Malaspina S., Magenta Biasina A. (2016). Magnetic resonance imaging and positron emission tomography in the diagnosis of neurodegenerative dementias. Funct. Neurol..

[9] Jie C.V.M.L., Treyer V., Schibli R., Mu L. (2021). Tauvid^TM^: The First FDA-Approved PET Tracer for Imaging Tau Pathology in Alzheimer’s Disease. Pharmaceuticals.

[10] Di Lazzaro V., Bella R., Benussi A., Bologna M., Borroni B., Capone F., Chen K.S., Chen R., Chistyakov A.V., Classen J. (2021). Diagnostic contribution and therapeutic perspectives of transcranial magnetic stimulation in dementia. Clin. Neurophysiol..

[11] Battaglia S., Nazzi C., Di Fazio C., Borgomaneri S. (2024). The role of pre-supplementary motor cortex in action control with emotional stimuli: A repetitive transcranial magnetic stimulation study. Ann. N. Y. Acad. Sci..

[12] Battaglia S., Nazzi C., Fullana M.A., di Pellegrino G., Borgomaneri S. (2024). ‘Nip it in the bud’: Low-frequency rTMS of the prefrontal cortex disrupts threat memory consolidation in humans. Behav. Res. Ther..

[13] Golde T.E. (2022). Disease-Modifying Therapies for Alzheimer’s Disease: More Questions than Answers. Neurotherapeutics.

[14] Sperling R.A., Jack C.R., Black S.E., Frosch M.P., Greenberg S.M., Hyman B.T., Scheltens P., Carrillo M.C., Thies W., Bednar M.M. (2011). Amyloid-related imaging abnormalities in amyloid-modifying therapeutic trials: Recommendations from the Alzheimer’s Association Research Roundtable Workgroup. Alzheimer’s Dement..

[15] Honig L.S., Sabbagh M.N., van Dyck C.H., Sperling R.A., Hersch S., Matta A., Giorgi L., Gee M., Kanekiyo M., Li D. (2024). Updated safety results from phase 3 lecanemab study in early Alzheimer’s disease. Alzheimer’s Res. Ther..

[16] Haeberlein S.B., Aisen P., Barkhof F., Chalkias S., Chen T., Cohen S., Dent G., Hansson O., Harrison K., von Hehn C. (2022). Two Randomized Phase 3 Studies of Aducanumab in Early Alzheimer’s Disease. J. Prev. Alzheimer’s Dis..

[17] Agarwal A., Gupta V., Brahmbhatt P., Desai A., Vibhute P., Joseph-Mathurin N., Bathla G. (2023). Amyloid-related Imaging Abnormalities in Alzheimer Disease Treated with Anti–Amyloid-β Therapy. RadioGraphics.

[18] Doran S.J., Sawyer R.P. (2024). Risk factors in developing amyloid related imaging abnormalities (ARIA) and clinical implications. Front. Neurosci..

[19] Choi K.S., Sunwoo L. (2022). Artificial Intelligence in Neuroimaging: Clinical Applications. Investig. Magn. Reson. Imaging.

[20] Jytzler J.A., Lysdahlgaard S. (2024). Radiomics evaluation for the early detection of Alzheimer’s dementia using T1-weighted MRI. Radiography.

[21] Matsoukas S., Scaggiante J., Schuldt B.R., Smith C.J., Chennareddy S., Kalagara R., Majidi S., Bederson J.B., Fifi J.T., Mocco J. (2022). Accuracy of artificial intelligence for the detection of intracranial hemorrhage and chronic cerebral microbleeds: A systematic review and pooled analysis. Radiol. Medica.

[22] Shafieioun A., Ghaffari H., Baradaran M., Rigi A., Eftekhar M.S., Shojaeshafiei F., Korani M.A., Hatami B., Shirdel S., Ghanbari K. (2025). Predictive power of artificial intelligence for malignant cerebral edema in stroke patients: A CT-based systematic review and meta-analysis of prevalence and diagnostic performance. Neurosurg. Rev..

[23] Marino L., Bilotta F. (2025). Artificial intelligence in traumatic brain injury: Brain imaging analysis and outcome prediction: A mini review. World J. Crit. Care Med..

[24] Sima D.M., Phan T.V., Van Eyndhoven S., Vercruyssen S., Magalhães R., Liseune A., Brys A., Frenyo P., Terzopoulos V., Maes C. (2024). Artificial Intelligence Assistive Software Tool for Automated Detection and Quantification of Amyloid-Related Imaging Abnormalities. JAMA Netw. Open.

[25] Roytman M., Mashriqi F., Al-Tawil K., Schulz P.E., Zaharchuk G., Benzinger T.L.S., Franceschi A.M. (2023). Amyloid-Related Imaging Abnormalities: An Update. Am. J. Roentgenol..

[26] Greenberg S.M., Vernooij M.W., Cordonnier C., Viswanathan A., Salman R.A.-S., Warach S., Launer L.J., Van Buchem M.A., Breteler M.M. (2009). Cerebral microbleeds: A guide to detection and interpretation. Lancet Neurol..

[27] Jeong S.Y., Suh C.H., Kim S.J., Lemere C.A., Lim J.-S., Lee J.-H. (2024). Amyloid-Related Imaging Abnormalities in the Era of Anti-Amyloid Beta Monoclonal Antibodies for Alzheimer’s Disease: Recent Updates on Clinical and Imaging Features and MRI Monitoring. Korean J. Radiol..

[28] Van Dyck C.H., Swanson C.J., Aisen P., Bateman R.J., Chen C., Gee M., Kanekiyo M., Li D., Reyderman L., Cohen S. (2023). Lecanemab in Early Alzheimer’s Disease. N. Engl. J. Med..

[29] Sims J.R., Zimmer J.A., Evans C.D., Lu M., Ardayfio P., Sparks J., Wessels A.M., Shcherbinin S., Wang H., Monkul Nery E.S. (2023). Donanemab in Early Symptomatic Alzheimer Disease: The TRAILBLAZER-ALZ 2 Randomized Clinical Trial. JAMA.

[30] Cummings J., Apostolova L., Rabinovici G.D., Atri A., Aisen P., Greenberg S., Hendrix S., Selkoe D., Weiner M., Petersen R.C. (2023). Lecanemab: Appropriate Use Recommendations. J. Prev. Alzheimer’s Dis..

[31] Rabinovici G., Selkoe D., Schindler S., Aisen P., Apostolova L., Atri A., Greenberg S., Hendrix S., Petersen R., Weiner M. (2025). Donanemab: Appropriate use recommendations. J. Prev. Alzheimer’s Dis..

[32] Barakos J., Purcell D., Suhy J., Chalkias S., Burkett P., Grassi C.M., Castrillo-Viguera C., Rubino I., Vijverberg E. (2022). Detection and Management of Amyloid-Related Imaging Abnormalities in Patients with Alzheimer’s Disease Treated with Anti-Amyloid Beta Therapy. J. Prev. Alzheimer’s Dis..

[33] Qiao Y., Chi Y., Zhang Q., Ma Y. (2023). Safety and efficacy of lecanemab for Alzheimer’s disease: A systematic review and meta-analysis of randomized clinical trials. Front. Aging Neurosci..

[34] Hampel H., Elhage A., Cho M., Apostolova L.G., Nicoll J.A.R., Atri A. (2023). Amyloid-related imaging abnormalities (ARIA): Radiological, biological and clinical characteristics. Brain.

[35] Hsu C.C., Sethi S.K., Haacke E.M. (2023). The Current State of Susceptibility-Weighted Imaging and Quantitative Susceptibility Mapping in Head Trauma. Neuroimaging Clin. N. Am..

[36] Genc O., Morrison M.A., Villanueva-Meyer J.E., Burns B., Hess C.P., Banerjee S., Lupo J.M. (2023). DeepSWI: Using Deep Learning to Enhance Susceptibility Contrast on T2*-Weighted MRI. J. Magn. Reson. Imaging.

[37] Liu S., Utriainen D., Chai C., Chen Y., Wang L., Sethi S.K., Xia S., Haacke E.M. (2019). Cerebral microbleed detection using Susceptibility Weighted Imaging and deep learning. NeuroImage.

[38] Yoon J., Gong E., Chatnuntawech I., Bilgic B., Lee J., Jung W., Ko J., Jung H., Setsompop K., Zaharchuk G. (2018). Quantitative susceptibility mapping using deep neural network: QSMnet. NeuroImage.

[39] Schmeel F.C. (2019). Variability in quantitative diffusion-weighted MR imaging (DWI) across different scanners and imaging sites: Is there a potential consensus that can help reducing the limits of expected bias?. Eur. Radiol..

[40] Sundaresan V., Arthofer C., Zamboni G., Dineen R.A., Rothwell P.M., Sotiropoulos S.N., Auer D.P., Tozer D.J., Markus H.S., Miller K.L. (2022). Automated Detection of Candidate Subjects with Cerebral Microbleeds Using Machine Learning. Front. Neurosci..

[41] Myung M.J., Lee K.M., Kim H.-G., Oh J., Lee J.Y., Shin I., Kim E.J., Lee J.S. (2021). Novel Approaches to Detection of Cerebral Microbleeds: Single Deep Learning Model to Achieve a Balanced Performance. J. Stroke Cerebrovasc. Dis..

[42] Momeni S., Fazlollahi A., Lebrat L., Yates P., Rowe C., Gao Y., Liew A.W.-C., Salvado O. (2021). Generative Model of Brain Microbleeds for MRI Detection of Vascular Marker of Neurodegenerative Diseases. Front. Neurosci..

[43] Aker L., Abandeh L., Abdelhady M., Aboughalia H., Vattoth S. (2022). Susceptibility-weighted Imaging in Neuroradiology: Practical Imaging Principles, Pearls and Pitfalls. Curr. Probl. Diagn. Radiol..

[44] Al-Masni M.A., Kim W.R., Kim E.Y., Noh Y., Kim D.H. (2020). Automated detection of cerebral microbleeds in MR images: A two-stage deep learning approach. NeuroImage Clin..

[45] Luo Y., Gao K., Fawaz M., Wu B., Zhong Y., Zhou Y., Haacke E.M., Dai Y., Liu S. (2024). Automatic detection of cerebral microbleeds using susceptibility weighted imaging and artificial intelligence. Quant. Imaging Med. Surg..

[46] Navab N., Hornegger J., Wells W.M., Frangi A.F. (2015). Medical Image Computing and Computer-Assisted Intervention—MICCAI 2015. Proceedings of the 18th International Conference.

[47] Isensee F., Jaeger P.F., Kohl S.A.A., Petersen J., Maier-Hein K.H. (2021). nnU-Net: A self-configuring method for deep learning-based biomedical image segmentation. Nat. Methods..

[48] Beheshti I., Sone D., Leung C.K., Advances of Artificial Intelligence in Neuroimaging (2025). Brain Sciences. https://www.embase.com/search/results?subaction=viewrecord&id=L2034339004&from=export.

[49] Maier O., Menze B.H., von der Gablentz J., Häni L., Heinrich M.P., Liebrand M., Winzeck S., Basit A., Bentley P., Chen L. (2017). ISLES 2015—A public evaluation benchmark for ischemic stroke lesion segmentation from multispectral MRI. Med. Image Anal..

[50] Bareja R., Ismail M., Martin D., Nayate A., Yadav I., Labbad M., Dullur P., Garg S., Tamrazi B., Salloum R. (2024). nnU-Net–based Segmentation of Tumor Subcompartments in Pediatric Medulloblastoma Using Multiparametric MRI: A Multi-institutional Study. Radiol. Artif. Intell..

[51] Lee H., Kim J., Lee S., Jung K., Kim W., Noh Y., Kim E.Y., Kang K.M., Sohn C., Lee D.Y. (2023). Detection of Cerebral Microbleeds in MR Images Using a Single-Stage Triplanar Ensemble Detection Network (TPE-Det). J. Magn. Reson. Imaging.

[52] Won S.Y., Kim J.-H., Woo C., Kim D.-H., Park K.Y., Kim E.Y., Baek S.-Y., Han H.J., Sohn B. (2024). Real-world application of a 3D deep learning model for detecting and localizing cerebral microbleeds. Acta Neurochir..

[53] Valverde J.M., Imani V., Abdollahzadeh A., De Feo R., Prakash M., Ciszek R., Tohka J. (2021). Transfer Learning in Magnetic Resonance Brain Imaging: A Systematic Review. J. Imaging.

[54] Kim H.E., Cosa-Linan A., Santhanam N., Jannesari M., Maros M.E., Ganslandt T. (2022). Transfer learning for medical image classification: A literature review. BMC Med. Imaging.

[55] Xie L., Yushkevich P.A., Das S.R., Wolk D.A., Gibson E. (2025). Utilizing Advanced Artificial Intelligence for Automated Detection and Segmentation of Amyloid-Related Imaging Abnormality (ARIA). Alzheimer’s Dement..

[56] Sima D., Phan T.V., Van Eyndhoven S., Vercruyssen S., Magalhães R., Maes C., Khan R., Guo J., Hughes R., Gabr R. (2024). Validation of Icobrain Aria—An AI-based Software Tool for Automated Detection and Quantification of Amyloidrelated Imaging Abnormalities. Neurology.

[57] FDA 510(k) Premarket Notification. https://www.accessdata.fda.gov/scripts/cdrh/cfdocs/cfpmn/pmn.cfm?ID=K240712.

[58] Marco M. FDA Clears Icobrain Aria, First AI Tool for Safer ARIA Detection in Alzheimer Treatment. https://www.neurologylive.com/view/fda-clears-icobrain-aria-first-ai-tool-safer-aria-detection-alzheimer-treatment.

[59] Cortechs ai Nets FDA Clearance for NeuroQuant 5.0 with Improved ARIA Quantification. https://appliedradiology.com/articles/cortechs-ai-nets-fda-clearance-for-neuroquant-5-0-with-improved-aria-quantification.

[60] Petrella J.R., Liu A.J., Wang L.A., Doraiswamy P.M. (2025). AI-Assisted Detection of Amyloid-related Imaging Abnormalities (ARIA): Promise and Pitfalls. AJNR Am.

[61] Salloway S., Wojtowicz J., Voyle N., Lane C.A., Klein G., Lyons M., Rossomanno S., Mazzo F., Bullain S., Barkhof F. (2025). Amyloid-Related Imaging Abnormalities (ARIA) in Clinical Trials of Gantenerumab in Early Alzheimer Disease. JAMA Neurol..

[62] Swanson C.J., Zhang Y., Dhadda S., Wang J., Kaplow J., Lai R.Y.K., Lannfelt L., Bradley H., Rabe M., Koyama A. (2021). A randomized, double-blind, phase 2b proof-of-concept clinical trial in early Alzheimer’s disease with lecanemab, an anti-Aβ protofibril antibody. Alzheimer’s Res. Ther..

[63] Wang H., Nery E.S.M., Ardayfio P., Khanna R., Svaldi D.O., Gueorguieva I., Shcherbinin S., Andersen S.W., Hauck P.M., Engle S.E. (2025). Modified titration of donanemab reduces ARIA risk and maintains amyloid reduction. Alzheimer’s Dement..

[64] Oakden-Rayner L., Dunnmon J., Carneiro G., Re C. (2020). Hidden stratification causes clinically meaningful failures in machine learning for medical imaging. Proceedings of the ACM Conference on Health, Inference, and Learning.

[65] Guan H., Liu M. (2022). Domain Adaptation for Medical Image Analysis: A Survey. IEEE Trans. Biomed. Eng..

[66] Momeni F., Shahbazi-Gahrouei D., Mahmoudi T., Mehdizadeh A. (2025). Transfer Learning and Neural Network-Based Approach on Structural MRI Data for Prediction and Classification of Alzheimer’s Disease. Diagnostics.

[67] Christodoulou R.C., Woodward A., Pitsillos R., Ibrahim R., Georgiou M.F. (2025). Artificial Intelligence in Alzheimer’s Disease Diagnosis and Prognosis Using PET-MRI: A Narrative Review of High-Impact Literature Post-Tauvid Approval. J. Clin. Med..

[68] Christodoulou R., Vamvouras G., Lorentzen L., Vassiliou E. (2025). Erythrocyte Load in Cerebrospinal Fluid Linked with Hippocampal Atrophy in Alzheimer’s Disease. J. Clin. Med..

[69] Christodoulou R.C., Vamvouras G., Petrou V., Papageorgiou P.S., Pitsillos R., Rivera L., Vassiliou E., Papageorgiou S.G., Solomou E.E., for the Alzheimer’s Disease Neuroimaging Initiative (2025). Cerebrospinal Fluid Erythrocyte Burden Amplifies the Impact of PTAU on Entorhinal Degeneration in Alzheimer’s Disease. Biomolecules.

[70] Lee Y.Y., Vaghari D., Burkhart M.C., Tino P., Montagnese M., Li Z., Zühlsdorff K., Giorgio J., Williams G., Chong E. (2024). Robust and interpretable AI-guided marker for early dementia prediction in real-world clinical settings. eClinicalMedicine.

[71] Christodoulou R., Christofi G., Pitsillos R., Ibrahim R., Papageorgiou P., Papageorgiou S.G., Vassiliou E., Georgiou M.F. (2025). AI-Based Classification of Mild Cognitive Impairment and Cognitively Normal Patients. J. Clin. Med..

[72] Sundaresan V., Arthofer C., Zamboni G., Murchison A.G., Dineen R.A., Rothwell P.M., Auer D.P., Wang C., Miller K.L., Tendler B.C. (2023). Automated detection of cerebral microbleeds on MR images using knowledge distillation framework. Front. Neurosci..

[73] Ali Z., Naz S., Yasmin S., Bukhari M., Kim M. (2023). Deep learning-assisted IoMT framework for cerebral microbleed detection. Heliyon.

[74] Smucny J., Shi G., Davidson I. (2022). Deep Learning in Neuroimaging: Overcoming Challenges with Emerging Approaches. Front. Psychiatry.

